# Diagnosis of genitourinary tuberculosis: detection of mycobacterial lipoarabinomannan and MPT-64 biomarkers within urine extracellular vesicles by nano-based immuno-PCR assay

**DOI:** 10.1038/s41598-023-38740-3

**Published:** 2023-07-18

**Authors:** Ekta Kamra, Tulika Prasad, Anam Rais, Bhawna Dahiya, Abhishek Sheoran, Aishwarya Soni, Suman Sharma, Promod K. Mehta

**Affiliations:** 1 Centre for Biotechnology, Maharshi Dayanand University, Rohtak, 124001 India; 2 Special Centre for Nano Science and Advanced Instrumentation Research Facility, Jawaharlal Nehru University, New Delhi, 110067 India; 3 Department of Statistics, Ramanujan College, University of Delhi, New Delhi, 110019 India; 4 Department of Biotechnology, Deenbandhu Chhotu Ram University of Science and Technology, Murthal, Sonipat, 131039 India; 5 Department of Microbiology, University of Health Sciences (UHS), Rohtak, 124001 India; 6 Department of Microbiology, Faculty of Allied Health Sciences, SGT University, Gurgaon, 122505 India

**Keywords:** Microbiology, Molecular biology, Diseases, Pathogenesis

## Abstract

We detected a cocktail of *Mycobacterium tuberculosis* lipoarabinomannan (LAM) and MPT-64 biomarkers within urine extracellular vesicles (EVs) of genitourinary TB (GUTB) patients by nano-based immuno-PCR (I-PCR) assay, i.e.*,* magnetic bead-coupled gold nanoparticle-based I-PCR (MB-AuNP-I-PCR) and compared the results with I-PCR and Magneto-ELISA. The size (s) of urine EVs ranged between 52.6 and 220.4 nm as analyzed by transmission electron microscopy (TEM) and nanoparticle tracking analysis. Functionalized AuNPs (coupled with detection antibodies/oligonucleotides) were characterized by UV–vis spectroscopy, TEM, ELISA, PCR, Atomic Force Microscopy and Fourier Transform Infrared spectroscopy, while conjugation of capture antibodies with MBs was validated by UV–vis spectroscopy and Magneto-ELISA. Our MB-AuNP-I-PCR exhibited sensitivities of 85% and 87.2% in clinically suspected (n = 40) and total (n = 47) GUTB cases, respectively, with 97.1% specificity in non-TB controls (n = 35). These results were further authenticated by the quantitative SYBR Green MB-AuNP-real-time I-PCR (MB-AuNP-RT-I-PCR). Concurrently, I-PCR and Magneto-ELISA showed sensitivities of 68.1% and 61.7%, respectively in total GUTB cases, which were significantly lower (*p* < 0.05–0.01) than MB-AuNP-I-PCR. Markedly, a wide range (400 fg/mL–11 ng/mL) of LAM+MPT-64 was quantified within urine EVs of GUTB cases by SYBR Green MB-AuNP-RT-I-PCR, which can assess the disease dynamics. This study will certainly improve the current algorithms used in GUTB diagnostics.

## Introduction

Before COVID-19 pandemic, tuberculosis (TB) was the foremost cause of deaths from a single infectious agent, ranking above HIV/AIDS but now, TB is the second infectious killer after COVID-19^1^. In 2021, approximately 1.4 million and 0.19 million deaths were reported among HIV-negative and HIV-positive TB infected individuals, respectively^1^. The COVID-19 pandemic had a negative impact on access to TB diagnosis, treatment and overall TB burden^1^. In 2021, India accounted for the highest TB burden (28%), followed by Indonesia (9.2%) and China (7.4%)^1^.Genitourinary TB (GUTB) is the second most reported form of extrapulmonary TB (EPTB) in developing countries like India and the third widespread form in low endemic countries^2^. The unusual clinical presentations, localization of disease at varied anatomical sites and paucibacillary nature of specimens make diagnosis of male/female GUTB a consternate challenge. To reduce the morbidity/mortality associated with GUTB, prompt and precise diagnosis is immediately required. In developing countries, smear microscopy is widely used but it lacks sensitivity as well as reproducibility^3^. Though culture identification of *Mycobacterium tuberculosis* (*Mtb*) from endometrial biopsies (EBs)/urine specimens of GUTB cases is considered to be the gold/reference standard, it generally exhibits low sensitivity on Lowenstein-Jensen (LJ) medium due to meager bacterial load within them^4,5^. Meanwhile, it has a long turnaround time of 4–6 weeks and needs expertise of skillful technicians^5^. Histopathological examination (HPE) is an important technique to diagnose GUTB^5^, albeit HPE is not pathognomonic except for the presence of acid-fast bacteria (AFB), since it cannot differentiate between GUTB and other granulomatous diseases^6,7^. Markedly, ultrasonography, computed tomography scan and intravenous urography are the main imaging modalities employed for both male/female GUTB diagnosis, while laparoscopy and hysterosalpingography are mostly used in diagnosing female GUTB^4,8^, yet no imaging technique alone is adequate for definite diagnosis.

Of note, it is tedious to collect GUTB specimens, e.g.*,* prostate/renal biopsies and EBs, since they involve a rigorous invasive procedure, it would be worthwhile to explore easily accessible specimens, such as urine, plasma/serum, etc.^4,6^. The key advantage of collecting urine over other biofluids is that it is easily available in copious amount and obtained in a non-invasive manner^9^. However, concentration of various biomarkers and biochemical constituents are quite variable in urine and of dynamic nature owing to different fluid intake, time of sample collection, age and different health status of the individuals^9^.

Extracellular vesicles (EVs), the membrane-bound liquid biopsies are present in various bodily fluids, including urine, serum and pleural/ascitic fluid^10,11^. The three main types of EVs (the nanostructures) are microvesicles (MVs), exosomes, and apoptotic bodies that are differentiated based on their biogenesis, size and function^12^. Markedly, exosomes (30–150 nm) or intraluminal vesicles arise from an endosomal route, MVs (100 nm–1 µm) are formed by pinching or direct outward budding of cell’s plasma membrane, and apoptotic bodies (50–5000 nm) are released by dying cells into the extracellular space^11–13^. Since neat urine contains a low concentration of *Mtb* biomarkers, EVs isolated from urine specimens of TB patients can concentrate those biomarkers and remove the contaminants, which leads to their better yield when evaluated by a sensitive method, e.g.*,* immuno-PCR (I-PCR)^14^. We detected individual lipoarabinomannan (LAM) and CFP-10 (Rv3874) within urine EVs of TB patients by I-PCR that exhibited superiority over ELISA^14^; LAM detection by I-PCR revealed better sensitivity than CFP-10 detection, yet these biomarkers were not evaluated in GUTB patients.

Moreover, a cocktail of *Mtb* MPT-64 (Rv1980c) and CFP-10 biomarkers was identified in TB patients by nano-based I-PCR assay, viz*.* magnetic bead-coupled gold nanoparticle-based I-PCR (MB-AuNP-I-PCR) with encouraging results^15^, albeit exclusive GUTB specimens were not assessed by this method. Strikingly, the utility of MBs (to couple capture antibodies) and functionalized AuNPs (conjugated with detection antibodies/ oligonucleotides) in MB-AuNP-I-PCR allows homogeneous attachment of antibodies to the target antigens in the liquid system, ensuring diminished background signals as well as sample matrix effect^16,17^. We also detected a cocktail of MPT-64 and ESAT-6 (Rv3875) in clinical specimens (EBs and urine) of GUTB patients by I-PCR with moderate sensitivity/specificity^5^. Concurrently, LAM and MPT-64 are contemplated as the prospective biomarkers to diagnose active TB within urine/urine EVs^5,14,18^. In order to further improve the diagnostic accuracy of I-PCR, we aimed to detect a cocktail of LAM+MPT-64 within urinary EVs of GUTB patients by MB-AuNP-I-PCR in this study and those results were directly compared with I-PCR and Magneto-ELISA assays. Additionally, we validated our MB-AuNP-I-PCR results with the quantitative SYBR Green MB-AuNP-real-time I-PCR (MB-AuNP-RT-I-PCR) assay.

## Results

In a preliminary study, amongst 10 urine specimens of clinically suspected GUTB cases, 6, 5 and 3 cases were found to be positive for LAM+MPT-64 detection by MB-AuNP-I-PCR, I-PCR and Magneto-ELISA, respectively. Therefore, an attempt was made to improve the sensitivity of MB-AuNP-I-PCR by detecting LAM+MPT-64 within urine EVs of GUTB patients. Prior to this, urine EVs were characterized by transmission electron microscopy (TEM) and nanoparticle tracking analysis (NTA).

### Characterization of urine EVs of GUTB cases

**Figure 1:**
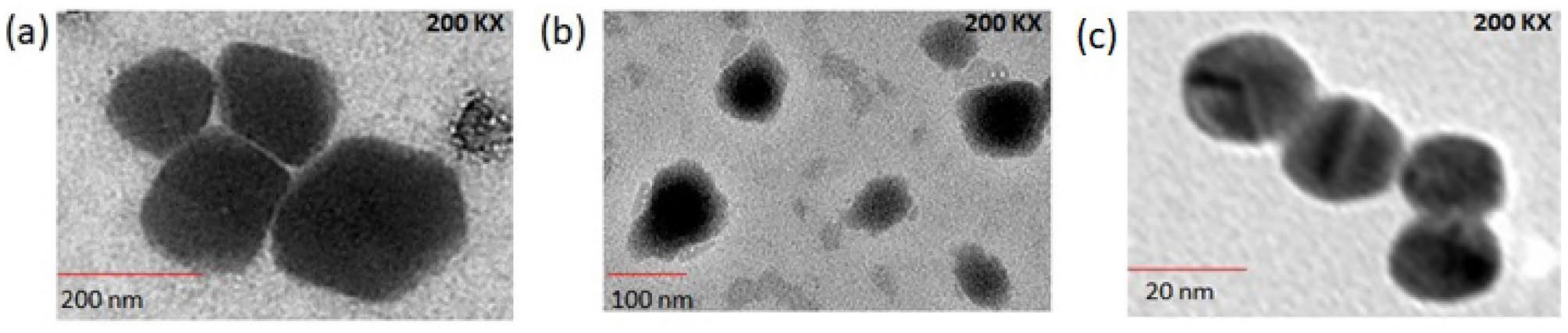
TEM images showing (**a**) size of urine EVs isolated from GUTB patients, (**b**) size of urine EVs isolated from healthy controls and (**c**) size of ~ 20 nm AuNPs.

TEM micrographs of urine EVs of GUTB cases showed that their size varied between 52.6 and 184 nm, whereas the size of urinary EVs of healthy controls varied from 79.4 to 137 nm (Fig. 1a,b). However, the mean particle size of urine EVs of GUTB cases ranged from 203 ± 81.8 to 220.4 ± 84 nm by NTA technique, in this study.

### Characterization of AuNPs and functionalized AuNPs (coupled with detection antibodies and oligonucleotides)

**Figure 2:**
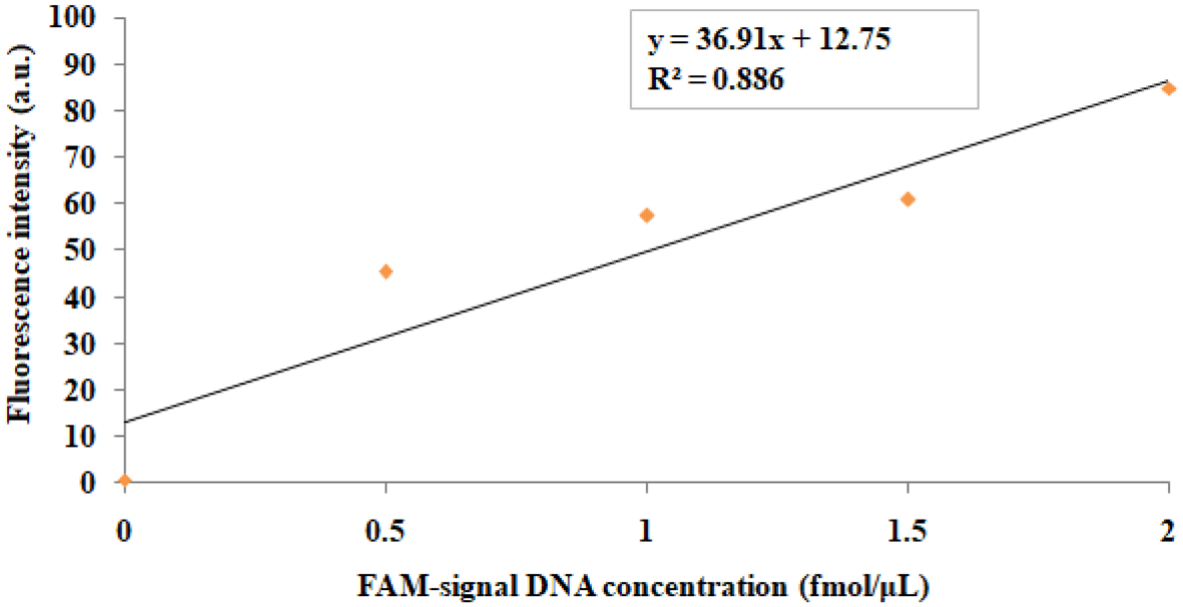
A standard curve was plotted between the fluorescence intensity vs. different concentrations (from 0.5 to 2 fmol/µL) of FAM-signal DNA. The regression equation was found to be y = 36.91x+12.75. The mean fluorescence intensity values were derived from two replicates in the graph. Each datum point represents the mean value of two independent experiments done in duplicates.

A single peak at 524.2 nm was observed for AuNPs by UV–vis spectroscopy (Supplementary Fig. 1a), while TEM images revealed that AuNPs were spherical with an average size of ~ 20 nm (Fig. 1c). Furthermore, the number of signal DNA molecules attached per AuNP in the functionalized AuNPs was determined by fluorescence spectrophotometry. First, a standard curve was plotted between the fluorescence intensity vs. different concentrations of fluorescein amidite-labelled signal DNA (FAM-signal DNA) (ranging from 0.5 to 2 fmol/µL) (Fig. 2). The replicates of ‘mean fluorescence intensity’ values for each datum point of this graph from two independent experiments are shown in Supplementary Table 1. The concentration of the released signal DNA in the functionalized AuNPs was found to be 1.17 fmol/µL, which yielded 0.468 pmol/µL after multiplying with a dilution factor of 1:400 and resulted in 73.3 signal DNA molecules per AuNP in the functionalized AuNPs.

Functionalized AuNPs coupled with detection antibodies and oligonucleotides were characterized by UV–vis spectroscopy, ELISA, PCR, TEM, Atomic Force Microscopy (AFM) as well as Fourier Transform Infrared (FTIR) spectroscopy. Notably, functionalized AuNPs showed a shift in the absorbance maxima peak of unbound AuNPs from 524.2 to 533.8 nm by UV–vis spectroscopy (Supplementary Fig. 1b), thereby, validating the conjugation of detection antibodies and oligonucleotides to AuNPs. Further, conjugation of detection antibodies to AuNPs was confirmed by ELISA (Supplementary Fig. 2a), wherein functionalized AuNPs (containing detection antibodies) showed a significantly higher mean optical density (OD) (*p* < 0.001), compared to unbound AuNPs. Concurrently, the conjugation of signal DNA to the functionalized AuNPs was validated by conventional PCR (Supplementary Fig. 2b), where a specific band of 76 bp was observed.

**Figure 3:**
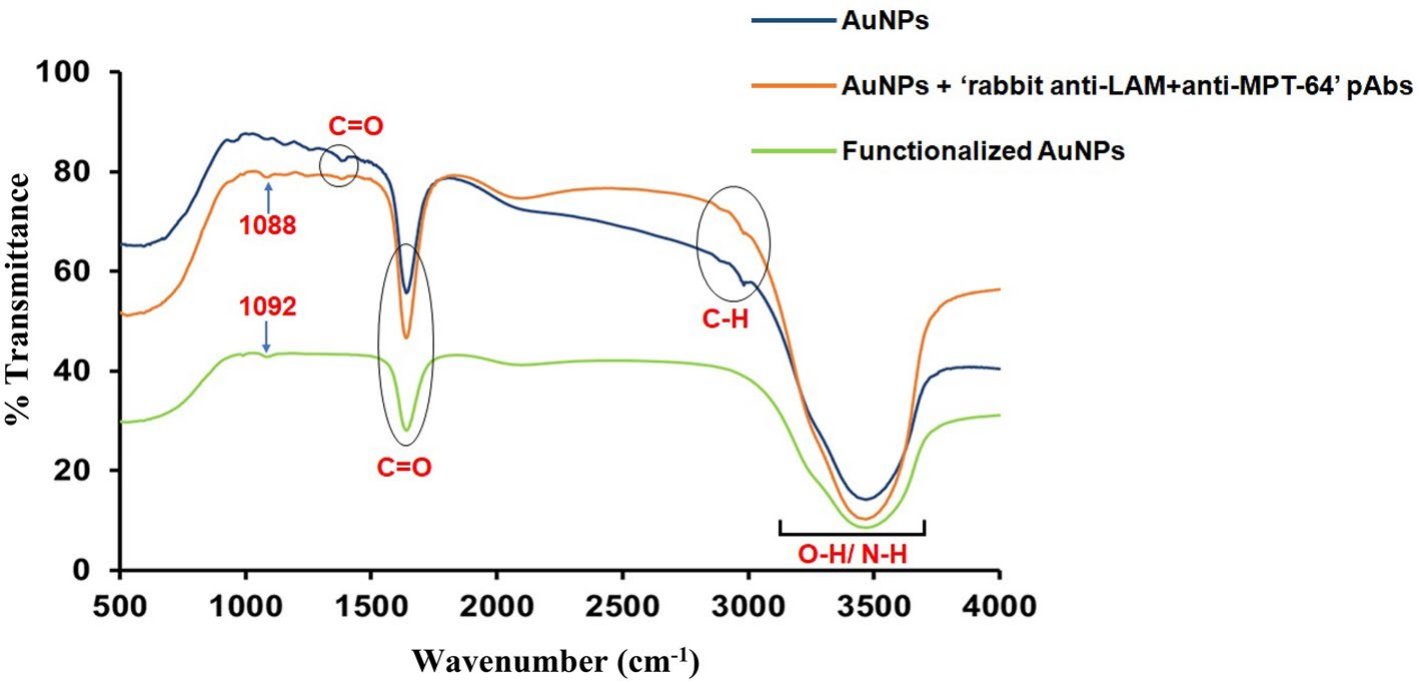
FTIR spectra of: AuNPs (Blue line), AuNPs conjugated with ‘rabbit anti-LAM+anti-MPT-64’ polyclonal antibodies (pAbs, detection antibodies) (Orange line) and functionalized AuNPs (conjugated with detection antibodies and oligonucleotides) (Green line).

FTIR spectroscopy detected changes in typical bands of AuNPs conjugated with detection antibodies and functionalized AuNPs. FTIR spectra before and after functionalization of AuNPs are depicted in Fig. 3, wherein a band of 3300–3600 cm^−1^ was observed due to O–H/N–H stretching in AuNPs, AuNPs+detection antibodies as well as functionalized AuNPs. However, broadening of the same band was noted in functionalized AuNPs (Green line), as compared with AuNPs (Blue line) and AuNPs+detection antibodies (Orange line), thus suggesting the stabilization of AuNPs through functionalization by breaking down of O–H/N–H bonds. Another characteristic band (corresponding to C-H stretching) at 2911 cm^−1^ and 2982 cm^−1^ was observed in AuNPs and AuNPs+detection antibodies, respectively, which was though missing in the functionalized AuNPs, since it was deformed after functionalization with detection antibodies/oligonucleotides. Moreover, the bands in AuNPs and AuNPs+detection antibodies at 1643 cm^−1^ and 1386 cm^−1^ indicated asymmetric and symmetric stretching of C=O bending vibrations, which was less intense in the functionalized AuNPs. Most of the bands disappeared for AuNPs between 1000 and 1500 cm^−1^ after functionalization. However, a band shift from 1088 to 1092 cm^−1^ was observed after functionalization of AuNPs. The changes in bands observed in FTIR spectra confirmed the stable conjugation of AuNPs with detection antibodies as well as in functionalized AuNPs.

**Figure 4:**
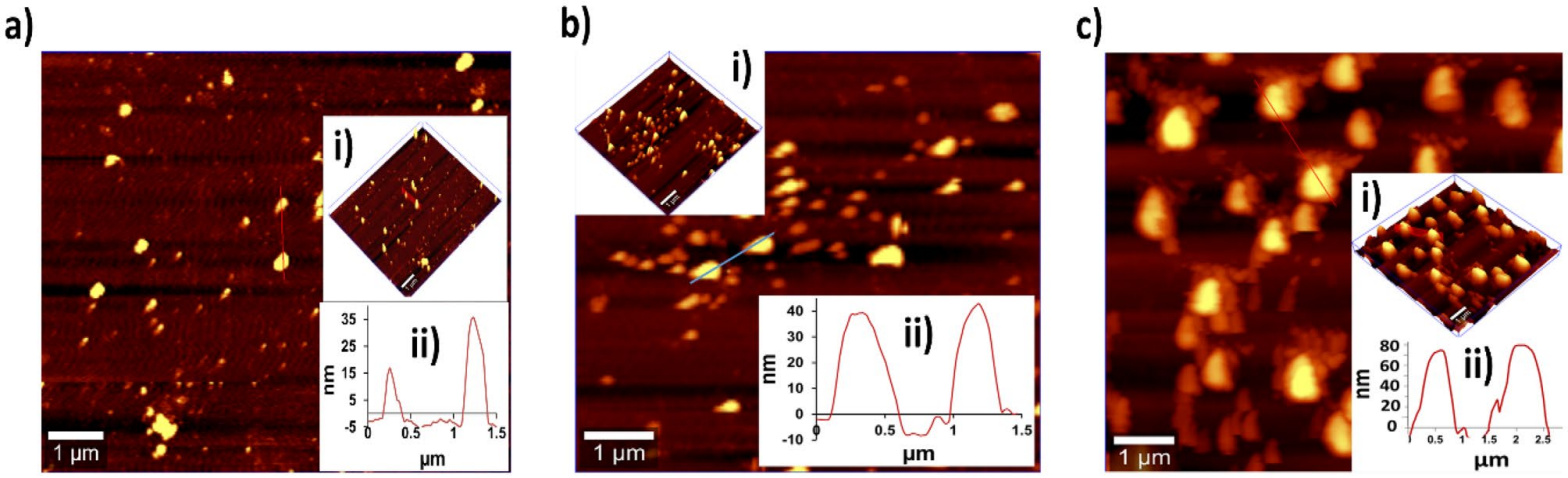
AFM images showing (**a**) AuNPs where inset (i) represents 3D topography and inset (ii) represents height distribution, (**b**) AuNPs conjugated with ‘rabbit anti-LAM+anti-MPT-64’ pAbs where inset (i) represents 3D topography and inset (ii) represents height distribution, while (**c**) functionalized AuNPs where inset (i) represents 3D topography and inset (ii) represents height distribution.

The 3D topography of AuNPs, AuNPs+detection antibodies and functionalized AuNPs are depicted in Fig. 4a–c (inset i). Interestingly, AFM images revealed a height distribution ranging between 15 and 35 nm for AuNPs (Fig. 4a inset ii) and mean height of 40 nm for AuNPs+detection antibodies (Fig. 4b inset ii). Concomitantly, the mean height was increased to 70 nm for functionalized AuNPs (Fig. 4c inset ii). Of note, an increase in height of AuNPs after conjugation with detection antibodies and functionalization indicated the formation and aggregation of larger particles.

### Validation of conjugation of capture antibodies with MBs

Coupling of capture antibodies, i.e., guinea pig anti-*Mtb* pAbs with MBs was confirmed by taking OD at 405 nm with Magneto-ELISA, which exhibited a significantly higher mean OD (*p* < 0.001) than unbound MBs (Supplementary Fig. 3a). Moreover, conjugation of capture antibodies to MBs was cross-validated by UV–vis spectroscopy at 270 nm, that showed a significantly higher mean OD (*p* < 0.001), compared with unbound MBs (Supplementary Fig. 3b).

### Limit of detection (LOD) for purified *Mtb* LAM+MPT-64 by MB-AuNP-I-PCR, SYBR Green MB-AuNP-RT-I-PCR, I-PCR and Magneto-ELISA

**Figure 5:**
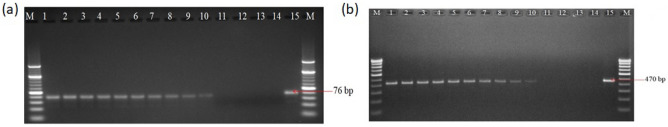
(**a**) LOD determination for the purified *Mtb* LAM+MPT-64 by MB-AuNP-I-PCR assay, wherein a 76 bp amplicon indicated a positive test on 4% agarose gel**:** Lane M, 20 bp ladder; lanes 1–12, serial ten-fold dilutions of the purified LAM+MPT-64 (from 1 µg/mL to 10 ag/mL); lane 13, I-PCR-negative control (no antigen coated, rest all the reagents added); lane 14, PCR-negative control (no template DNA); lane 15, PCR-positive control (signal DNA, 1 ng/mL). The experiments were performed thrice and one of the representative figures has been shown. (**b**) LOD determination for the purified *Mtb* LAM+MPT-64 by I-PCR assay, where a 470 bp amplicon indicated a positive test on 2% agarose gel: Lane M, 100 bp ladder; lanes 1–12, serial ten-fold dilutions of the purified LAM+MPT-64 (from 1 µg/mL to 10 ag/mL); lane 13, I-PCR-negative control (no antigen coated, rest all the reagents added); lane 14, PCR-negative control (no template DNA); and lane 15, PCR-positive control (reporter biotinylated DNA, 1 ng/mL). The experiments were performed thrice and one of the representative figures has been shown.

**Figure 6:**
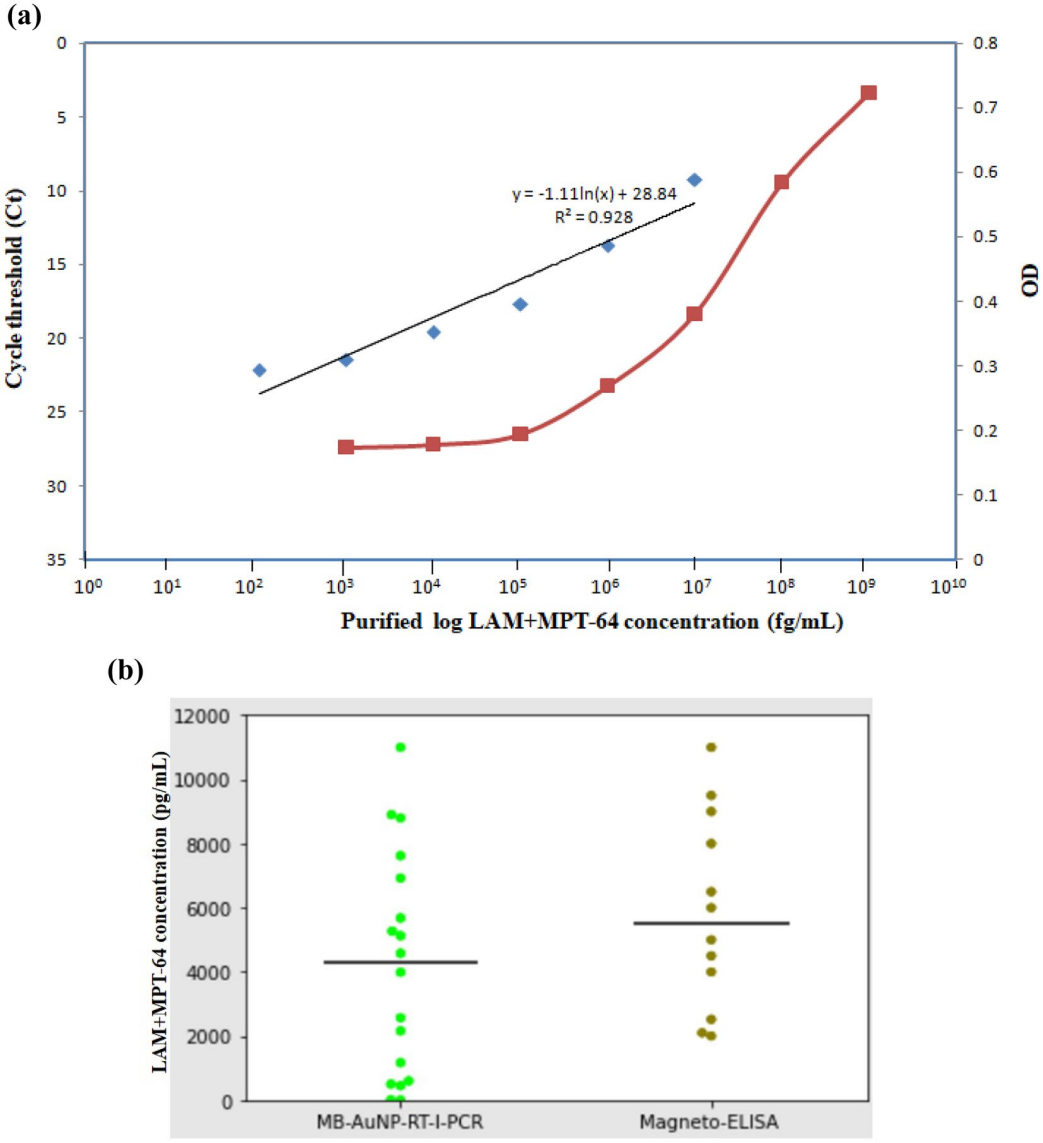
(**a**) The standard curve(s) for the purified LAM+MPT-64 by MB-AuNP-RT-I-PCR (from 100 fg/mL to 10 ng/mL) and Magneto-ELISA (from 100 pg/mL to 1 µg/mL)**:** a correlation coefficient was found to be 0.928 for MB-AuNP-RT-I-PCR and the corresponding regression equation was Ct = − 1.11 × ln (conc.)+28.84. The mean Ct (for MB-AuNP-RT-I-PCR) and OD (for Magneto-ELISA) values were derived from two replicates. Each datum point represents the mean value of two independent experiments done in duplicates. (**b**). Scatter diagram for MB-AuNP-RT-I-PCR and Magneto-ELISA, in which more variability [coefficient of variation (CV) of 80.6 vs. 50.9%] in LAM+MPT-64 concentration was observed for MB-AuNP-RT-I-PCR, compared with Magneto-ELISA. Black horizontal lines indicate the median value for both MB-AuNP-RT-I-PCR and Magneto-ELISA.

The schematic representations for MB-AuNP-I-PCR/MB-AuNP-RT-I-PCR and I-PCR/Magneto-ELISA are depicted in Supplementary Fig. 4a,b, respectively. We obtained an LOD of 1 fg/mL for purified *Mtb* LAM+MPT-64 (in buffer) by MB-AuNP-I-PCR and I-PCR (Fig. 5a,b). Interestingly, when urine EVs of healthy individuals (n = 5) were spiked with the purified LAM+MPT-64, a similar LOD of 1 fg/mL was obtained by MB-AuNP-I-PCR thus inferring no sample matrix effect, whereas I-PCR showed a higher LOD of 10 fg/mL (data not shown), inferring the presence of a sample matrix effect. The threshold cycle (Ct) values were determined by setting fluorescence in the exponential phase of amplification curves for SYBR Green MB-AuNP-RT-I-PCR. The standard curve was plotted between the Ct values vs. log purified LAM+MPT-64 concentrations (Fig. 6a), where the final detection range of LAM+MPT-64 was found to be 100 fg/mL–10 ng/mL by MB-AuNP-RT-I-PCR with an LOD of 45 fg/mL. On the contrary, Magneto-ELISA displayed a final detection range between 100 pg/mL and 1 µg/mL with an LOD of 100 pg/mL (Fig. 6a). The replicates of mean Ct and OD values for each datum point of this graph from two independent experiments are presented in Supplementary Table 2. Further, LODs of 60 fg/mL and 400 pg/mL were attained by MB-AuNP-RT-I-PCR and Magneto-ELISA, respectively, within urinary EVs (of a healthy individual) spiked with the purified LAM+MPT-64 (Supplementary Fig. 5), implying a marginal sample matrix effect with MB-AuNP-RT-I-PCR, as compared to Magneto-ELISA.

### LAM+MPT-64 detection within urine EVs of GUTB patients by MB-AuNP-I-PCR/I-PCR and Magneto-ELISA

We attained sensitivities of 100% [95% confidence interval (CI) 59–100%] and 85% (95% CI 70.2–94.3%) by MB-AuNP-I-PCR in urine EVs of seven confirmed and 40 clinically suspected GUTB patients, respectively (Table 1), against the composite reference standard (CRS) [a combination of clinical features, imaging, smear/culture, GeneXpert, IS*6110* PCR and response to anti-tubercular therapy (ATT)]. Remarkably, MB-AuNP-I-PCR revealed significantly higher (*p* < 0.05–0.01) sensitivities than I-PCR/Magneto-ELISA in both clinically suspected and total GUTB cases. Amongst 35 non-TB controls, only one and three cases showed false-positive results, demonstrating specificities of 97.1% (95% CI 85.1–99.9%) and 91.4% (95% CI 76.9–98.2%) by MB-AuNP-I-PCR and I-PCR/Magneto-ELISA, respectively (Table 1). Representative examples of GUTB cases and non-TB controls evaluated by MB-AuNP-I-PCR based on LAM+MPT-64 detection in urine EVs are depicted in Supplementary Fig. 6.

**Table 1:** Sensitivity and specificity of MB-AuNP-I-PCR, I-PCR and Magneto-ELISA at 95% CI based on LAM+MPT-64 detection within urine EVs of GUTB patients/non-TB controls, against CRS as the reference standard.

Categories of GUTB	MB-AuNP-I-PCR			I-PCR			Magneto-ELISA		
	(+)	% Sensitivity	% Specificity	(+)	% Sensitivity	% Specificity	(+)	% Sensitivity	% Specificity
Confirmed (n = 7)	7	100 (59–100)		6	85.7 (42.1–99.6)		5	71.4 (29–96.3)	
Clinically suspected (n = 40)	34	†85 (70.2–94.3)		26	†65 (48.3–79.4)		24	†60 (43.3–75.1)	
Total GUTB (n = 47)	41	*87.2 (74.3–95.2)		32	*68.1 (52.9–80.9)		29	*61.7 (46.4–75.5)	
Non-TB controls (n = 35)	1		97.1 (85.1–99.9)	3		91.4 (76.9–98.2)	3		91.4 (76.9–98.2)

Significant differences (*p* < 0.05–0.01) were noted between the sensitivities of †clinically suspected and *total GUTB cases by MB-AuNP-I-PCR, compared with I-PCR and Magneto-ELISA, using exact symmetry test.

n, number of samples; (+), positive cases; GUTB, genitourinary tuberculosis.

### LAM+MPT-64 detection within urine EVs of GUTB patients by a quantitative SYBR Green MB-AuNP-RT-I-PCR

Notably, a wide range of 400 fg/mL–11 ng/mL of LAM+MPT-64 concentration was detected within urinary EVs of GUTB cases by MB-AuNP-RT-I-PCR, while a narrow range (2–11 ng/mL) of the same was detected by Magneto-ELISA. To determine the variability of the observations, scatter plots of MB-AuNP-RT-I-PCR and Magneto-ELISA were drawn (Fig. 6b), wherein a higher coefficient of variation (CV) was attained for MB-AuNP-RT-I-PCR, as compared to Magneto-ELISA (80.6 vs. 50.9%), demonstrating detection of a wider range of LAM+MPT-64 concentrations in urinary EVs of GUTB patients by MB-AuNP-RT-I-PCR than Magneto-ELISA. Subsequently, the mean Ct and OD value (of two replicates) obtained for each patient by the quantitative MB-AuNP-RT-I-PCR and Magneto-ELISA are shown in Table 2. For MB-AuNP-RT-I-PCR, the mean Ct values lesser than NC+3SD (26.20) were considered to be positive for GUTB cases, whereas the values greater than 26.20 were considered as non-TB subjects. Concurrently, the mean OD values greater than ‘mean OD of non-TB control group+2SD’ (0.189) were considered to be positive for GUTB cases by Magneto-ELISA, while the values lesser than 0.189 were considered as non-TB subjects. This displayed a sensitivity of 85.7% (95% CI 63.7–96.9%) and specificity of 95% (95% CI, 75.1–99.9%) by MB-AuNP-RT-I-PCR in 21 clinically suspected GUTB cases and 20 non-TB controls, respectively (Table 3), which were comparable to MB-AuNP-I-PCR results.

**Table 2:** Replicates of Ct and OD values for MB-AuNP-RT-I-PCR and Magneto-ELISA, respectively, for individual GUTB/non-TB control subjects.

Group		MB-AuNP-RT-I-PCR			Magneto-ELISA		
		Ct1	Ct2	Mean Ct	OD1	OD2	Mean OD
Clinically suspected GUTB cases (n = 21)	1	9.42	9.3	9.36	0.362	0.363	0.3625
	2	10.3	10.25	10.275	0.326	0.321	0.3235
	3	10.6	11	10.8	0.312	0.309	0.3105
	4	11.2	11.4	11.3	0.168	0.165	0.1665
	5	12	11.9	11.95	0.302	0.301	0.3015
	6	13.8	13.6	13.7	0.172	0.174	0.173
	7	19.5	19	19.25	0.178	0.179	0.1785
	8	11.68	11.7	11.69	0.152	0.156	0.154
	9	12.45	12.48	12.465	0.298	0.294	0.296
	10	15.8	15.5	15.65	0.275	0.278	0.2765
	11	30.21	30.19	30.2	0.166	0.167	0.1665
	12	13.3	13	13.15	0.278	0.277	0.2775
	13	10.1	10.25	10.175	0.171	0.172	0.1715
	14	15.5	15.8	15.65	0.286	0.284	0.285
	15	30.18	30.16	30.17	0.176	0.179	0.1775
	16	12.8	12.84	12.82	0.281	0.283	0.282
	17	12.1	12.21	12.155	0.289	0.287	0.288
	18	11.49	11.62	11.555	0.172	0.172	0.172
	19	21.2	21.35	21.275	0.271	0.273	0.272
	20	15.78	15.85	15.815	0.329	0.327	0.328
	21	30.17	30.21	30.19	0.17	0.178	0.174
Non-TB controls (n = 20)	1	37.27	37.26	37.265	0.174	0.182	0.178
	2	27.48	27.49	27.485	0.179	0.176	0.1775
	3	31.71	31.75	31.73	0.178	0.165	0.1715
	4	29.07	29.17	29.12	0.187	0.172	0.1795
	5	28.69	28.59	28.64	0.182	0.179	0.1805
	6	32.56	32.68	32.62	0.181	0.178	0.1795
	7	21.18	21.3	21.24	0.209	0.198	0.2035
	8	34.26	34.22	34.24	0.167	0.178	0.1725
	9	29.01	29.13	29.07	0.178	0.175	0.1765
	10	31.06	30.96	31.01	0.195	0.162	0.1785
	11	34.09	34.21	34.15	0.181	0.177	0.179
	12	33.36	33.23	33.295	0.176	0.179	0.1775
	13	28.89	29.03	28.96	0.162	0.169	0.1655
	14	29.36	29.25	29.305	0.196	0.186	0.191
	15	36.69	36.82	36.755	0.166	0.179	0.1725
	16	38.25	38.14	38.195	0.158	0.176	0.167
	17	29.96	30.12	30.04	0.178	0.182	0.18
	18	30.03	29.88	29.955	0.134	0.167	0.1505
	19	34.25	34.37	34.31	0.125	0.146	0.1355
	20	33.6	33.46	33.53	0.135	0.116	0.1255

From these data, the mean Ct values lesser than NC+3SD (26.20) were considered to be positive for GUTB cases by MB-AuNP-RT-I-PCR, whereas the mean Ct values greater than 26.20 were considered as negative for non-TB controls. Meanwhile, the mean OD values greater than ‘mean OD of non-TB control group+2SD’ (0.189) were considered to be positive for GUTB cases by Magneto-ELISA, while the mean OD values lesser than 0.189 were considered as negative for non-TB controls.

**Table 3:** Sensitivity and specificity of MB-AuNP-RT-I-PCR and Magneto-ELISA at 95% CI based on LAM+MPT-64 detection within urinary EVs of clinically suspected GUTB patients/non-TB controls, against CRS as the reference standard.

Categories of GUTB	MB-AuNP-RT-I-PCR			Magneto-ELISA		
	( +)	% Sensitivity	% Specificity	( +)	% Sensitivity	% Specificity
Clinically suspected (n = 21)	18	85.7 (63.7–96.9)		12	57.1 (34–78.2)	
Non-TB controls (n = 20)	1		95 (75.1–99.9)	2		90 (68.3–98.7)

n, number of samples; ( +), positive cases; GUTB, genitourinary tuberculosis.

## Discussion

Diagnosis of GUTB is an off-putting challenge. In this study, we detected a cocktail of *Mtb* LAM and MPT-64 biomarkers within urine EVs of GUTB patients by MB-AuNP-I-PCR assay. We utilized the ‘total exosome isolation reagent’ to isolate urine EVs from GUTB cases, as employed in a previous study^14^. Urine EVs are usually isolated by ultracentrifugation and sucrose gradient centrifugation^19,20^, yet these methods have a prolonged turnaround time and provide low yield of EVs. TEM micrographs of urine EVs of GUTB patients and healthy controls showed their size between 52.6 and 184 nm (Fig. 1a,b). This finding is well in consensus with a previous report^21^, wherein the size of urine EVs of prostate cancer patients and healthy controls (isolated by high-speed centrifugation) ranged from 20 to 230 nm as revealed by TEM. Concomitantly, the mean particle size of urine EVs of GUTB patients assessed by NTA technique ranged between 203 ± 81.8 and 220.4 ± 84 nm, in this study. However, NTA has several limitations as it requires a vibration-free environment and analysis of less-viscous specimens^22^, though we evaluated the size of urine EVs by both NTA and TEM analysis.

Furthermore, slightly polydispersed and spherical ~ 20 nm AuNPs were observed by TEM (Fig. 1c), which were subsequently functionalized with ‘rabbit anti-LAM+MPT-64’ pAbs (detection antibodies) and oligonucleotides. This resulted in a red shift from 524.2 nm (corresponded to a characteristic surface plasmon resonance band of AuNPs) to 533.8 nm in the UV–vis spectrum, confirming the conjugation of detection antibodies/oligonucleotides to AuNPs (Supplementary Fig. 1b). This finding is in agreement with the previous reports on UV–vis spectroscopic characterization of functionalized AuNPs to detect purified *Mtb* ESAT-6 and CFP-10 proteins by AuNP-RT-I-PCR/MB-AuNP-I-PCR assays^17,23,24^. While detecting interleukin-3 and stem cell factor by AuNP-RT-I-PCR, Potuckova et al.^25^ documented a shift of 5–10 nm in wavelength when AuNPs were conjugated with detection antibodies/oligonucleotides. Notably, the number of signal DNA molecules attached per 20 nm AuNP was determined to be ~ 73 molecules/AuNP in the functionalized AuNPs (Fig. 2), which is in concurrence with the previous reports^26,27^, wherein 75–100 molecules of signal DNA were attached per AuNP of 30 nm size.

The conjugation of ‘anti-LAM+MPT-64’ antibodies to the functionalized AuNPs was also confirmed by ELISA (Supplementary Fig. 2a), wherein a significantly higher OD (*p* < 0.001) was observed for functionalized AuNPs, as compared to unbound AuNPs. Meanwhile, the functionalized AuNPs revealed a 76 bp amplicon by PCR (Supplementary Fig. 2b), validating the conjugation of signal DNA to the functionalized AuNPs. Likewise, while detecting purified *Mtb* CFP-10 and CFP-10+MPT-64 by AuNP-RT-I-PCR/MB-AuNP-I-PCR, attachment of detection antibodies and oligonucleotides to the functionalized AuNPs was confirmed by ELISA and PCR, respectively^15,23^.

FTIR spectroscopy was utilized to characterize the functionalized AuNPs in the present study (Fig. 3), wherein a band between 3300 and 3600 cm^−1^ was observed owing to O–H/N–H stretching in AuNPs, AuNPs+detection antibodies and functionalized AuNPs. However, broadening of the same band was noted in the functionalized AuNPs (Fig. 3), suggesting the stabilization of AuNPs through functionalization by breaking down the O–H/N–H bonds. Similarly, FTIR spectra of AuNPs revealed two major peaks for OH and C=O stretching at 3307 and 1635 cm^−1^, respectively, whereas a medium peak was observed at 1365 cm^−1^ (corresponding to C-O stretching vibration)^28^. The same phenomenon was also documented in FTIR spectra of AuNPs coupled with ‘mouse anti-HBsAg (hepatitis B surface antigen)’ mAbs^29^, wherein the characteristic peak observed with unbound AuNPs at 3435 cm^−1^ (owing to OH stretching vibrations) disappeared when AuNPs were coupled with detection antibodies. Additionally, three peaks were observed at 1650 cm^−1^/616 cm^−1^ and 3335 cm^−1^ due to N–H deformation as well as N–H stretching vibrations, respectively, thus cross-validating the coupling of the ‘anti-HBsAg’ mAbs with AuNPs^29^.

Simultaneously, AFM was utilized to evaluate the surface topography and height distribution of AuNPs, AuNPs+detection antibodies and functionalized AuNPs (Fig. 4), wherein an increase in height indicated the conjugation of detection antibodies/oligonucleotides to functionalized AuNPs. Unlike unbound AuNPs, a similar increase in height was observed by AFM when AuNPs were conjugated with bovine serum albumin (BSA)^30,31^. Similarly, UV–vis spectra of phenylalanine-carbon quantum dots (Phe-CQDs), the biocompatible non-toxic nanomaterials exhibited two absorption peaks at 250 and 340 nm, implying the characteristic absorption of aromatic π system^32^. Moreover, AFM of the Phe-CQDs revealed 3D topography with a mean height of 5 nm, while FTIR revealed broad absorption bands between 3100 and 3500 cm^−1^ due to O–H and N–H vibrations^32^.

The conjugation of capture antibodies to MBs was confirmed by Magneto-ELISA as well as UV–vis spectroscopy (Supplementary Fig. 3a,b), the same techniques were utilized to confirm the coupling of capture antibodies with MBs in previous papers^15,17,24^. Afterwards, we determined an LOD of 1 fg/mL for the purified *Mtb* LAM+MPT-64 by MB-AuNP-I-PCR in buffer (Fig. 5a) as well as urine EVs (of healthy individuals) spiked with the purified antigen and demonstrated no sample matrix effect. Similarly, LODs of 1 fg/mL and 10 fg/mL were obtained without any sample matrix effect for purified *Mtb* CFP-10+MPT-64 and ESAT-6, respectively by MB-AuNP-I-PCR in both buffer and saliva samples spiked with purified antigen^15,17^. However, both I-PCR/Magneto-ELISA in the present study exhibited sample matrix effect, which was evident from higher LODs obtained in urine EVs (of healthy individuals) spiked with the purified LAM+MPT-64 than that obtained in buffer. Concomitantly, HIV-1 p24 Gag protein was detected by MB-AuNP-RT-I-PCR with < 200 CD4+T cells/μL in plasma of HIV-1-infected men^33^. Staphylococcal enterotoxin B has also been detected up to 269 fg/mL in various food samples by magnetic microparticle-AuNP-RT-I-PCR^16^.

Markedly, CD4+cells were detected up to 50 cells/µL in blood samples of AIDS patients by Magneto-ELISA^34^. In contrast to synthetic MBs utilized for Magneto-ELISA and MB-AuNP-I-PCR in the present study, Oh et al.^35^ employed silica-coated Fe_3_O_4_ nanoclusters (‘magnetic nanobeads’) to design a magnetic nanozyme-linked immunosorbent assay that attained an LOD of 100 pg/mL for detecting influenza A virus. Conversely, biogenic magnetosomes of *Magnetospirillum gryphiswaldense* were utilized in Magneto I-PCR to detect the purified HBsAg within human sera with an LOD of 320 pg/mL^36^. However, biogenic magnetosomes have some limitations owing to limited tolerance towards detergents and lower stability of their biological membranes^15,17^.

We obtained a high sensitivity (87.2%) and specificity (97.1%) by MB-AuNP-I-PCR based on LAM+MPT-64 detection in urine EVs of 47 total GUTB patients and 35 non-TB controls, respectively with respect to CRS (Table 1). Further, the sensitivity attained by MB-AuNP-I-PCR was significantly higher (*p* < 0.05–0.01) than I-PCR (68.1%) and Magneto-ELISA (61.7%). We also corroborated our MB-AuNP-I-PCR results with the quantitative SYBR Green MB-AuNP-RT-I-PCR that exhibited almost equivalent results (85.7% sensitivity and 95% specificity) to diagnose clinically suspected GUTB patients in urine EVs (Table 3). While comparing LAM+MPT-64 detection in urine EVs of GUTB cases by MB-AuNP-I-PCR in this study with that of CFP-10+MPT-64 detection by MB-AuNP-I-PCR in EPTB specimens (pleural/ascitic fluids, pus, etc.)^15^, LAM+MPT-64 detection revealed a higher sensitivity (87.2 vs. 78.1%) than the latter. This could be due to detection of a cocktail of LAM+MPT-64, wherein LAM (instead of CFP-10) is considered a more favorable biomarker for TB detection within urine/urine EVs^14,37^. Moreover, LAM+MPT-64 concentration was probably enhanced within urine EVs of GUTB cases, as compared to neat EPTB specimens utilized in a previous study^15^. Similarly, while comparing LAM+MPT-64 detection in urine EVs of GUTB cases by MB-AuNP-I-PCR with that of LAM detection in urine EVs of total EPTB cases by conventional I-PCR, detection of LAM+MPT-64 exhibited better sensitivity (87.2 vs. 67.9%) and specificity (97.1 vs. 92.7%) than the latter^14^. This was most likely owing to LAM+MPT-64 detection (rather than LAM alone) within urine EVs, different nature of specimens (GUTB vs. total EPTB) in the two studies and more importantly, the usage of MB-AuNP-I-PCR, which is relatively a more precise method than I-PCR to detect biomarkers in clinical specimens^15^.

Of note, there are two distinct populations of EVs released from *Mtb* infected macrophages, e.g., those enclosing the host EVs cell markers (CD63/CD9) and the *Mtb* markers, such as lipoglycans (LAM) and lipoproteins (LpqH) that regulate both innate and acquired immune responses^38,39^. Similarly, *Mycobacterium bovis* BCG-infected human THP-1 and murine J774 macrophages were shown to release exosomes containing LAM, LpqH and Ag85 complex^40^. Moreover, the release of mycobacterial EVs (MEVs) containing LAM/LpqH depends on *Mtb*’s viability, entailing that active *Mtb* replication is essential for MEVs generation^38,39^, thus suggesting that LAM/LpqH detection within MEVs may indicate active TB disease. Humoral responses to MEVs (containing LAM) with sera of HIV-negative pulmonary TB patients were also documented by ELISA and immunoblot techniques^41^. Comparable to LAM+MPT-64 detection in urine EVs, *Mtb* CFP-2 (Rv2376c), MPT-32 (Rv1860), MPT-64, and BfrB (Rv3841) peptides were detected within serum exosomes of TB patients by advanced multiple reaction monitoring-mass spectrometry (MRM-MS)^42^, though this method is not appropriate to diagnose TB in field settings. Additionally, *Mtb* GlnA1 (Rv2220), GarA (Rv1827), GroES (Rv3418c) and DnaK (Rv0350) peptides were detected within serum exosomes of latent TB individuals by MRM-MS^43^. Identification of circulating platelet-derived EVs by flow cytometry has also been documented in COVID-19 patients^44^.

Remarkably, the enhanced sensitivity (87.2%) attributed by MB-AuNP-I-PCR in this study could also be due to dual amplification, i.e*.,* usage of multivalent AuNPs that release multiple signal DNAs per antibody molecule, whereas PCR imparts another degree of amplification^15^. Moreover, high specificity (97.1%) attained by MB-AuNP-I-PCR was probably due to vigorous washing and elimination of unbound antigens/antibodies in the liquid system by using an external magnetic field to suppress background noise and sample matrix effect^15,33^. Likewise, Kim et al.^33^ exhibited high sensitivity (99%) and specificity (100%) by MB-AuNP-RT-I-PCR based on p24 Gag protein detection within plasma samples of HIV-1 infected individuals.

Our quantitative SYBR Green MB-AuNP-RT-I-PCR can also monitor the disease dynamics, since a dynamic range of LAM+MPT-64 (400 fg/mL to 11 ng/mL) was detected within urinary EVs of GUTB patients (Fig. 6b). Both LAM and MPT-64 represent biomarkers for active *Mtb* replication, their enhanced levels within urine/urinary EVs may reflect the progression of active GUTB disease^5,14,39^. We previously demonstrated diminished MPT-64+PstS1 (Rv0934) concentrations by RT-I-PCR in clinical specimens of pulmonary TB patients on therapy and probably the decreased bacterial load^45^. Similarly, this work can be further extended to evaluate the regression of disease by MB-AuNP-RT-I-PCR within urinary EVs of GUTB patients on ATT. However, MB-AuNP-RT-I-PCR requires expensive real-time PCR equipment and skilled technicians, while MB-AuNP-I-PCR is comparatively a less costly method that may be utilized to diagnose GUTB cases on a routine basis.

To conclude, we developed an MB-AuNP-I-PCR assay (liquid format) for detecting a cocktail of *Mtb* LAM+MPT-64 in urine EVs of GUTB patients, which demonstrated high sensitivity (~ 87%) and specificity (~ 97%), as compared to I-PCR and Magneto-ELISA. Indeed, a high diagnostic accuracy acquired by MB-AuNP-I-PCR superseded the sensitivity of the WHO guidelines^46^ for high-priority target product profiles (≥ 80% sensitivity and ≥ 98% specificity for EPTB diagnosis including GUTB) and almost matched the specificity to deliberate a new diagnostic test. To the best of our knowledge, this is the first report to detect LAM+MPT-64 in urine EVs of GUTB cases by MB-AuNP-I-PCR with promising results. After validating MB-AuNP-I-PCR data with a larger sample size from varied epidemiological settings and reducing the cost, this test may be translated into an attractive diagnostic kit.

## Methodology

### Reagents

Purified LAM (NR-14848), MPT-64 (NR-44102), rabbit anti-LAM pAbs (NR-13821) and guinea pig anti-*Mtb* CDC1551 pAbs (NR-13818) were received as generous gifts from BEI Resources, ATCC, Manassas, VA, USA, while rabbit anti-MPT-64 pAbs were purchased from Abcam (Cambridge, UK). ‘Total exosome isolation reagent’ from urine (catalogue # 4484452) was purchased from Thermo Fisher Scientific, whereas RoboStrips were purchased from AJ Roboscreen, Leipzig, Germany. BSA, sulfo-SMCC [succinimidyl 4-(N-maleimidomethyl)-cyclohexane-1-carboxylate)] and 1,4-dithiothreitol (DTT) were procured from Merck KGaA (Darmstadt, Germany).

### Collection of urine specimens from GUTB/non-TB control subjects

Early morning mid-stream urine specimens (~ 5 mL) were obtained from the strongly suggestive male/female GUTB patients and non-TB control subjects (by taking their written informed consent) in the hospital associated with Urology/Obstetrics and Gynaecology Department, UHS, Rohtak. This proposal was approved by the Institutional Human Ethics Committees (IHECs) of Maharshi Dayanand University, Rohtak (IHEC/19/02 and IHEC/2021/306) and our research was performed as per the guidelines stated by the Declaration of Helsinki for human participants. Urine samples were centrifuged at 2000×*g* for 30 min at 4 °C to remove the cells/debris and the supernatants were stored at – 20 ºC until further use.

**Figure 7:**
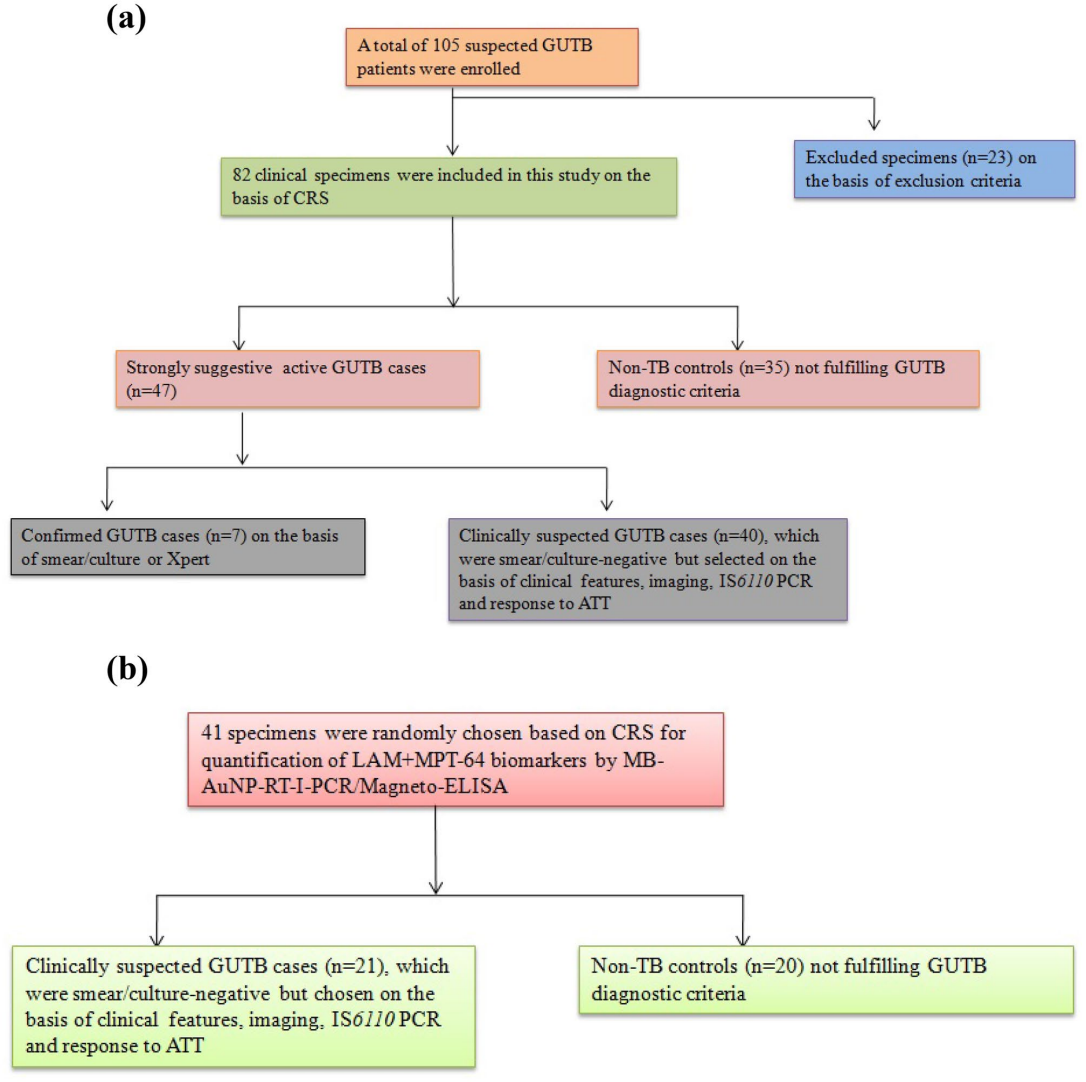
(**a**) Flowchart for grouping of the study participants (GUTB patients/non-TB controls) evaluated for LAM+MPT-64 detection by MB-AuNP-I-PCR/I-PCR and Magneto-ELISA. (**b**) Flowchart for grouping of the study participants (GUTB patients/non-TB controls) evaluated for LAM+MPT-64 detection by SYBR Green MB-AuNP-RT-I-PCR and Magneto-ELISA.

Urine samples of GUTB patients (n = 47) were broadly categorized as: (i) Confirmed GUTB cases (n = 7), which were positive for AFB by smear microscopy using Ziehl–Neelsen staining, culture identification for *Mtb* on LJ medium or positive GeneXpert^5^, (ii) Clinically suspected GUTB cases (n = 40), which were all smear-negative/culture-negative but chosen on the basis of clinical features, imaging, IS*6110* PCR and response to ATT. In addition, (iii) Non-TB controls (n = 35) that comprised patients with urinary tract infection of non-TB origin (n = 11) and menstrual irregularities (n = 24). However, patients already on ATT and individuals with proven multi-drug resistant/extensively-drug resistant-TB cases at the time of specimen collection were excluded^5^. Furthermore, individuals with symptoms suggestive of pulmonary TB and other EPTB types but not of GUTB, HIV-co-infected individuals, diabetic/cancer patients and individuals not responding to ATT after eight weeks of therapy were excluded. The flowchart of the study participants divided into different groups for evaluating urine EVs of GUTB cases and non-TB controls for LAM+MPT-64 detection by MB-AuNP-I-PCR/I-PCR and Magneto-ELISA has been depicted in Fig. 7a.

Moreover, 41 patients were randomly chosen (21 clinically suspected GUTB cases and 20 non-TB controls) and their urine EVs were analysed for LAM+MPT-64 detection using a quantitative SYBR Green MB-AuNP-RT-I-PCR and Magneto-ELISA (Fig. 7b).

### Isolation of urine EVs

Urine supernatants of GUTB cases/non-TB controls were removed from refrigerator and brought to room temperature. Equal volumes of urine supernatants and ‘total exosome isolation reagent’ were mixed until the solution became homogeneous^14^.The reaction mixtures were incubated at room temperature for 1 h followed by centrifugation at 10,000×*g* at 4 °C for 1 h. EVs present in pellets were resuspended in 50 μL of sterile PBS.

### Characterization of urine EVs

The protein content of EVs was determined by taking absorbance at 280 nm with Nanodrop TM 2000 spectrophotometer. The particle size/count of urinary EVs was determined by Nanosight NS300 NTA (Malvern Panalytical Application Lab, New Delhi). The size and shape of urinary EVs were also analyzed using Talos F200X TEM (Thermo Fischer; Sophisticated Analytical Instrumentation Facility, All India Institute of Medical Sciences, New Delhi), wherein 2 μL of freshly prepared EVs were dropped on a carbon coated grid and air dried. Those grids were stained with 2% phosphotungstic acid solution and subsequently dried before imaging.

### Preparation of biotinylated reporter DNA

Biotinylated reporter DNA was prepared by PCR amplification of *bla* gene of pUC19 plasmid DNA using 5′-biotin-GTCGTTTGGTATGGCTTC-3′ forward primer and 5′-CCTTCCTGTTTTTGCTCAC-3′ reverse primer and the amplified product was purified by a gel extraction kit^5^ and stored at 4 °C until further use.

### LAM+MPT-64 detection within urine EVs of GUTB patients/non-TB controls by I-PCR

A cocktail of LAM+MPT-64 was detected by I-PCR as detailed by Kamra et al.^5^ with little modifications. RoboStrip wells were coated with 50 μL of optimally diluted urine EVs (containing 200 μg/mL of protein) in the coating buffer (0.05 M carbonate buffer, pH 9.6), which translated to 8.4 × 10^11^–11.5 × 10^11^ particles/mL with a mean particle size ± SD of 203 ± 81.8 to 220.4 ± 84 nm. To determine LOD of the purified LAM+MPT-64, serial ten-fold dilutions [from 1 μg/mL to 10 attogram (ag)/mL] were prepared in the coating buffer and 50 μL of different diluents were coated on RoboStrip wells. All the urinary EVs of GUTB cases/non-TB control subjects were run in triplicates to ensure the reproducibility of results. RoboStrips were incubated overnight at 4 °C followed by washing with PBS containing 0.05% Tween-20 (PBST, washing buffer) at room temperature for 1 min under orbital shaking (400–500 rpm). Next, RoboStrip wells were blocked with a blocking buffer (3% BSA in PBST) at 37 °C for 2 h followed by washing and addition of 50 µL of ‘rabbit anti-LAM (1:500)+anti-MPT-64 (1:1000)’ pAbs. Rest of the steps were same as described in a previous paper^5^.

### Synthesis and characterization of AuNPs

AuNPs (~ 20 nm) were synthesized by chemical reduction^15^. To validate the preparation of AuNPs, the colloidal solution was scanned between 400 and 700 nm in UV–vis spectroscopy (UV-1800) and the OD was recorded. The size and shape of AuNPs were analyzed using a 200 kV TEM, while particle counts in AuNPs were determined by NTA technique. For TEM analysis, samples were prepared by placing a few drops of AuNPs on the grid and subsequently dried before imaging.

### Activation of capture DNA

Briefly, disulphide bonds of 1 nM solution of 100 μL thiolated DNA (5′-[C6Thiol] TTTTTTTTTTTTTTTGCTTGTCTCGTAAGTTGAGATTTCGCTATGCACGGTCCTT-3′) were activated, desalted and purified using the centrifugal filters as per the manufacturer’s instructions^17^ and stored at − 20 °C till further use.

### Functionalization of AuNPs with ‘rabbit anti-LAM+anti-MPT-64’ pAbs (detection antibodies) and oligonucleotides

Briefly, 500 μL of AuNPs (4 × 10^9^ particles/mL) were mixed with 500 μL of ‘rabbit anti-LAM (1:500)+anti-MPT-64 (1:1000)’ pAbs (1:1, v/v) and incubated at room temperature for 45 min on a dancing shaker^15^.To this, 100 μL of activated capture DNA was added and incubated at room temperature for 30 min, which was followed by the addition of 10% Tween-20, 2 M NaCl (in PBS) as well as 10% BSA, and incubated at room temperature for 30 min. The excess of capture DNA was removed by cold centrifugation at 12,000 rpm for 15 min and the particles were resuspended in 500 μL of PBS. Next, 5 pM of signal DNA (5′-CTGCGACGATCTACCATCGACGTACCAGGTCGGTTGAAGGACCGTGCATAGCGAAATCTCAACTTACGAGACAAGC-3′) was added to the residue followed by incubation at 37 °C for 1 h for hybridization, while rest of the steps were same as detailed in a previous paper^15^. Functionalized AuNPs were resuspended in PBS and stored at 4 °C till further use^17^. Particle counts in the functionalized AuNPs were found to be 2 × 10^9^ particles/mL by NTA.

To determine the number of signal DNA molecules attached per AuNP, functionalized AuNPs were prepared using FAM-signal DNA molecules^26^, and rest of the steps were identical for preparing functionalized AuNPs using unlabeled signal DNA^15^. The non-hybridized FAM-signal DNA molecules were removed by centrifugation at 12,000 rpm for 15 min, whereas the FAM-signal DNA molecules attached with functionalized AuNPs were dehybridized by heating at 95 °C for 15 min. The concentration of the released oligonucleotides was determined by fluorescence spectrophotometer (Perkin Elmer Spectrofluorimeter LS 55, Singapore) with excitation and emission wavelengths at 485 and 528 nm, respectively and interpolation from a standard linear calibration curve (Fig. 2) plotted between the fluorescence intensity vs. different FAM-signal DNA concentrations (from 0.5 to 2 fmol/µL). Markedly, FAM-signal DNA dehybridized from the functionalized AuNPs was diluted 1:400 (in PBS) prior to analysis. The average number of signal DNA molecules attached per AuNP was determined by dividing the signal DNA molar concentration with AuNP concentration in the functionalized AuNPs.

### Characterization of functionalized AuNPs

Functionalized AuNPs were characterized by monitoring a shift in the maximum absorbance peak^17^ with UV–vis spectroscopy. To validate the conjugation of ‘rabbit anti-LAM+anti-MPT-64’ pAbs with AuNPs in the functionalized AuNPs, indirect ELISA was employed^23^. Further, to confirm the coupling of signal DNA to the functionalized AuNPs, PCR was employed using forward (5′-CTGCGACGATCTACCAT-3′) and reverse primers (5′-GCTTGTCTCGTA AGTTGA-3′)^23^.

The presence of specific functional groups in AuNPs, AuNPs+detection antibodies and functionalized AuNPs (conjugated with detection antibodies/oligonucleotides) were also confirmed by recording the FTIR spectra with Varian 7000 FTIR spectrometer between 650 and 4000 cm^−1^^32^. Moreover, the surface mapping of AuNPs, AuNPs+detection antibodies and functionalized AuNPs were carried out using AFM (WITec Instrument, GmbH, Germany), wherein the samples were prepared by dropping them onto a mica substrate and dried at 37 °C^32^.

### Conjugation of capture antibodies (guinea pig anti-*Mtb* pAbs) with MBs

Activation of MBs by sulfo-SMCC and antibody reduction by DTT was performed simultaneously as described previously^17^. Later, MBs were resuspended in 500 μL of PBS and kept at 4 °C till further use. Coupling of capture antibodies with MBs was confirmed by taking ODs at 270 nm and 405 nm with UV–vis spectroscopy and Magneto-ELISA, respectively^15,17^.

### LAM+MPT-64 detection within urine EVs of GUTB patients/non-TB controls by MB-AuNP-I-PCR, SYBR Green MB-AuNP-RT-I-PCR and Magneto-ELISA

First, LODs of the purified LAM+MPT-64 were determined by MB-AuNP-I-PCR, SYBR Green MB-AuNP-RT-I-PCR and Magneto-ELISA. For that, serial ten-fold dilutions of antigen (from 1 µg/mL to 10 ag/mL in PBS) were prepared for MB-AuNP-I-PCR, while the range (s) were 10 ng/mL to 100 fg/mL and 1 µg/mL to 1 pg/mL for SYBR Green MB-AuNP-RT-I-PCR and Magneto-ELISA, respectively. Subsequently, 50 µL of MBs coupled with guinea pig anti-*Mtb* pAbs (1:1000) was taken in eppendorf tubes, followed by washing with ‘washing buffer’ (using an external magnetic field) and addition of 200 µL of blocking buffer (5% BSA in PBS). After thorough washing, 50 µL of different diluents of the purified LAM+MPT-64 or optimally diluted urine EVs (containing 200 µg/mL of protein) of GUTB patients/non-TB controls were added. All the clinical samples were run in triplicates for MB-AuNP-I-PCR (and the corresponding Magneto-ELISA) and in duplicates for SYBR Green MB-AuNP-RT-I-PCR (and the corresponding Magneto-ELISA). Reaction mixtures were incubated at room temperature for 1 h with constant shaking to avoid settling of MBs. MBs were thoroughly washed with ‘washing buffer’ and 50 µL of functionalized AuNPs were added. Reaction mixtures were again incubated at room temperature for 1 h with constant shaking followed by washing, which were resuspended in 100 µL of distilled water and heated at 95 °C for 15 min. After cooling to room temperature, an external magnetic field was applied and 10 µL of supernatant was taken to evaluate the released signal DNA by PCR^17^.

For MB-AuNP-RT-I-PCR, all the steps were same as for MB-AuNP-I-PCR except that real-time PCR (instead of PCR) was employed at the terminal end and USB® VerQuest™ SYBR® Green qPCR Mastermix was used as per the manufacturer's instructions^5^. Moreover, background control and negative control (NC) were included, which contained all the components except for ‘no reporter DNA’ and ‘no LAM+MPT-64’, respectively. Rest of the steps were same as described in a previous study^5^. For Magneto-ELISA, all the initial steps were like MB-AuNP-I-PCR but instead of adding functionalized AuNPs, ‘rabbit anti-LAM (1:500)+anti-MPT-64 (1:1000)’ pAbs and goat anti-rabbit IgG alkaline phosphatase (1:1000) were consecutively added after thorough washing at each step using an external magnetic field. Later, all the steps were followed as detailed in a previous paper^17^.

To determine the sample matrix effect by MB-AuNP-I-PCR, MB-AuNP-RT-I-PCR, I-PCR and Magneto-ELISA, urine EVs of healthy individuals pre-tested at various dilutions for the absence of LAM/MPT-64 were spiked with the purified LAM+MPT-64 (1 μg/mL) and the LODs were determined by preparing serial tenfold dilutions of the same^15,16^.

### Statistical analyses

The mean OD of clinical samples from the control group+2SD was used as a cutoff value for positive GUTB cases by Magneto-ELISA^17^, whereas LOD for LAM+MPT-64 by MB-AuNP-RT-I-PCR was calculated by NC+3SD^5^. Meanwhile, the presence or absence of a specific band indicates the presence/absence of antigen for I-PCR (470 bp) and MB-AuNP-I-PCR (76 bp)^14,15^. Sensitivity and specificity of MB-AuNP-I-PCR, I-PCR and Magneto-ELISA were determined at 95% CI, against CRS as the reference standard^5^. Comparison of MB-AuNP-I-PCR vs. I-PCR/Magneto-ELISA was made using the exact symmetry test, while comparisons of ODs between the different groups were made using the 2-sample *t*-test with equal variances^15^. The statistical software STATA/SE version 14.2 was employed for the analyses. A value of *p* < 0.05 was considered statistically significant.

## Supplementary Information

Supplementary Figures.

Supplementary Tables.

## Data Availability

All data generated or analysed during this study are included in the supplementary information files.
