# Supplementary material for: Diagnosis of genitourinary tuberculosis: detection of mycobacterial lipoarabinomannan and MPT-64 biomarkers within urine extracellular vesicles by nano-based immuno-PCR assay
Source: Sci Rep. 2023 Jul 18;13:11560. doi: 10.1038/s41598-023-38740-3 (PMC10354090; doi:10.1038/s41598-023-38740-3)
Supplement: Supplementary file 1 — Supplementary Figures. [file 41598_2023_38740_MOESM1_ESM.docx]

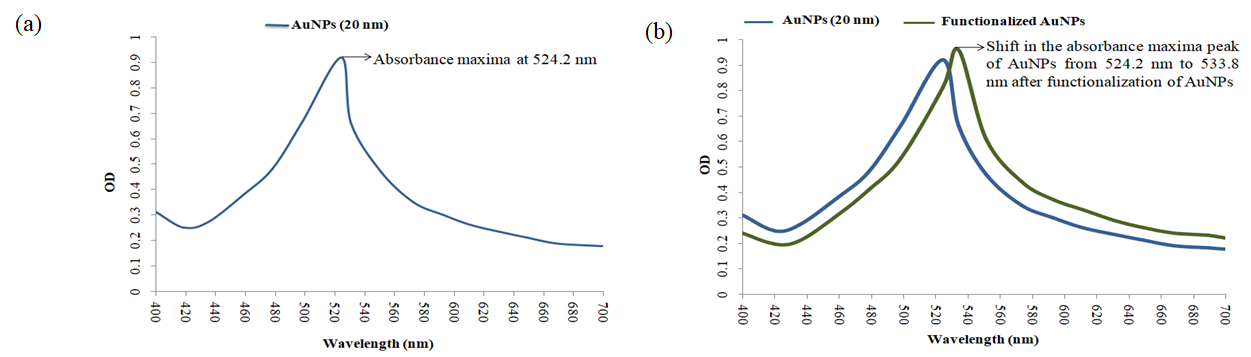


**Supplementary Figure 1 (a).** Absorbance spectra of 20 nm AuNPs in the UV-vis spectrum ranged between 400 to 700 nm with an absorbance maximum at 524.2 nm. **(b).** Overlay of UV-vis absorbance spectra for 20 nm AuNPs (524.2 nm) and functionalized AuNPs (533.8 nm).


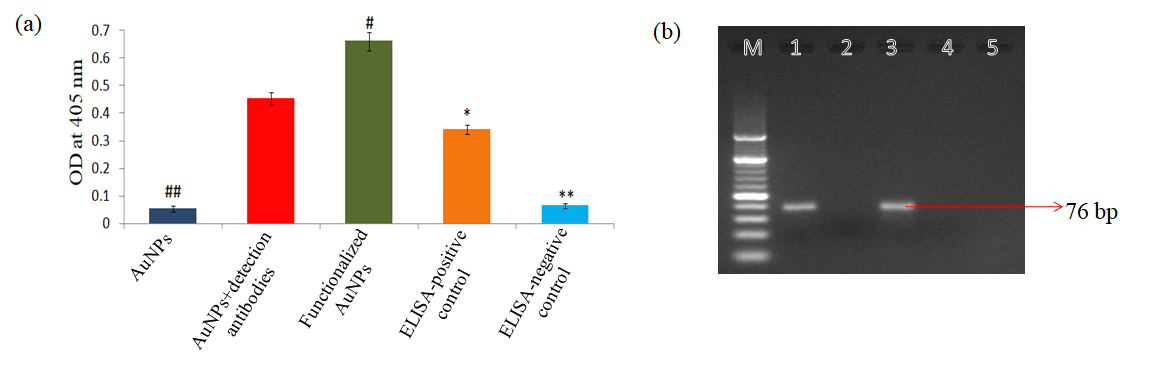


**Supplementary Figure 2 (a).** Conjugation of detection antibodies (‘rabbit anti-LAM+anti-MPT-64’ pAbs) to the functionalized AuNPs by ELISA at 405 nm: Bar diagram revealed that mean ^#^OD±SD of the functionalized AuNPs was significantly higher (*p*<0.001) than ^##^AuNPs alone. Similarly, mean ^*^OD±SD of ELISA-positive control (no AuNPs, LAM+MPT-64 coated, ‘rabbit anti-LAM+anti-MPT-64’ pAbs added) was significantly higher (*p*<0.05) than ^**^ELISA-negative control (no AuNPs, LAM+MPT-64 coated but no ‘rabbit anti-LAM+anti-MPT-64’ pAbs added) (done in duplicates). **(b).** Conjugation of signal DNA to the functionalized AuNPs by PCR analyzed on 4% agarose gel showing a 76 bp amplified product: lane M, 20 bp ladder; lane 1, functionalized AuNPs; lane 2, AuNPs+ ‘rabbit anti-LAM+anti-MPT-64’ pAbs+capture DNA but no signal DNA added; lane 3, PCR-positive control (signal DNA, 1 ng/mL); lane 4, PCR-negative control (no signal DNA); lane 5, PCR grade water only. The experiments were repeated twice and one of the representative figures has been shown.


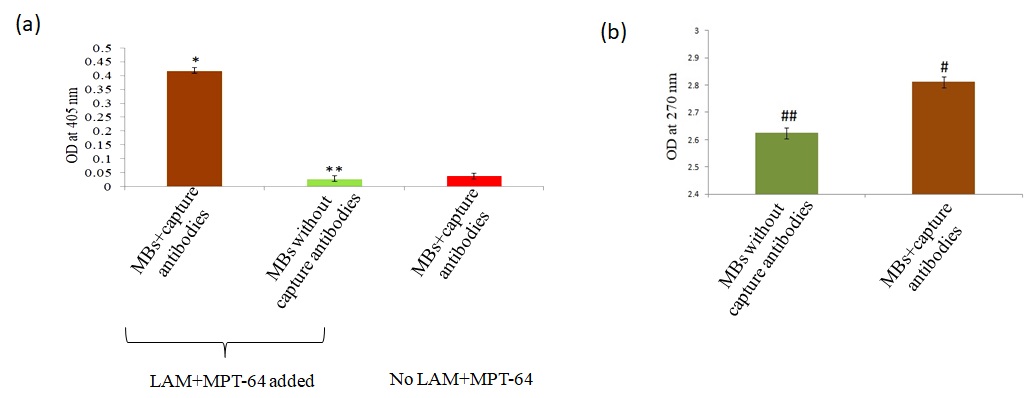


**Supplementary Figure 3 (a).** Conjugation of capture antibodies (guinea pig anti-*Mtb* pAbs) with MBs by Magneto-ELISA at 405 nm: Bar diagram revealed that *mean OD±SD of MBs+capture antibodies was significantly higher (*p*<0.001) than **unbound MBs (done in duplicates). Moreover, antigen control (no antigen coated, rest all the reagents added) revealed a negligible OD, thus further authenticating conjugation of MBs+capture antibodies. **(b).** Bar diagram revealed that ^#^mean OD±SD of MBs+capture antibodies was significantly higher (*p*<0.001), compared with ^##^MBs alone by UV-vis spectroscopy at 270 nm (done in duplicates).

**(a)**


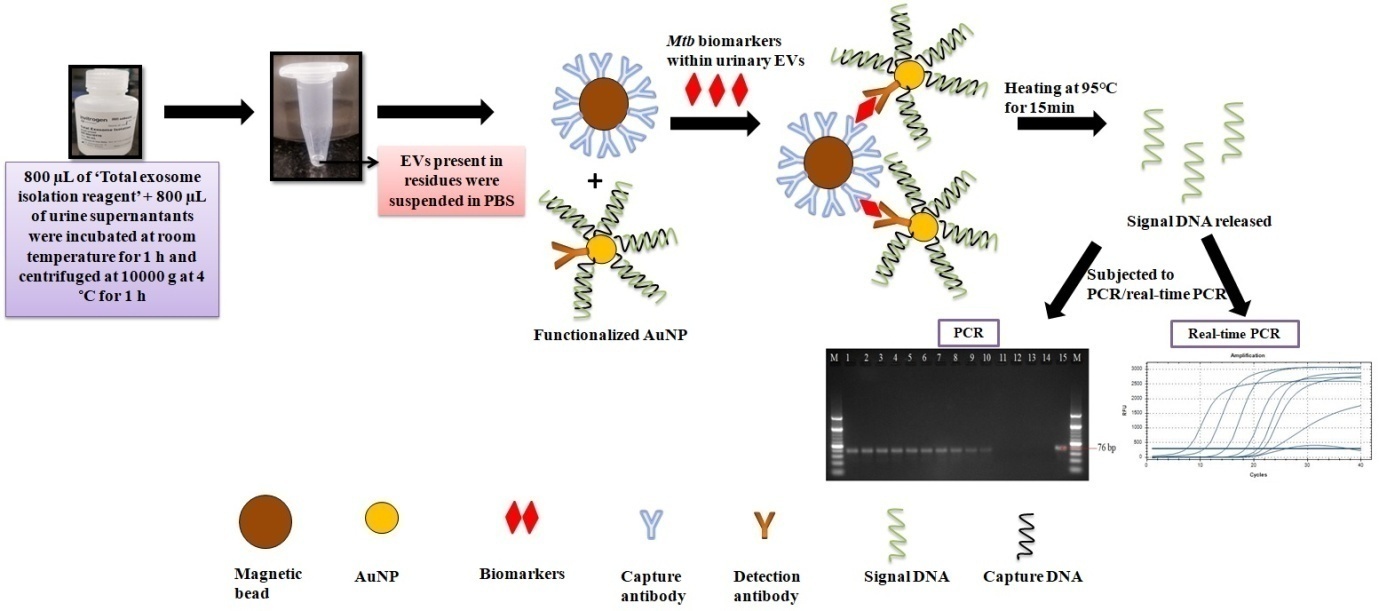


**Supplementary Figure 4 (a).** The schematic representation of MB-AuNP-I-PCR (PCR at the terminal end) and SYBR Green MB-AuNP-RT-I-PCR (real-time PCR at the terminal end) for *Mtb* LAM+MPT-64 detection within urine EVs of GUTB patients.

**(b)**


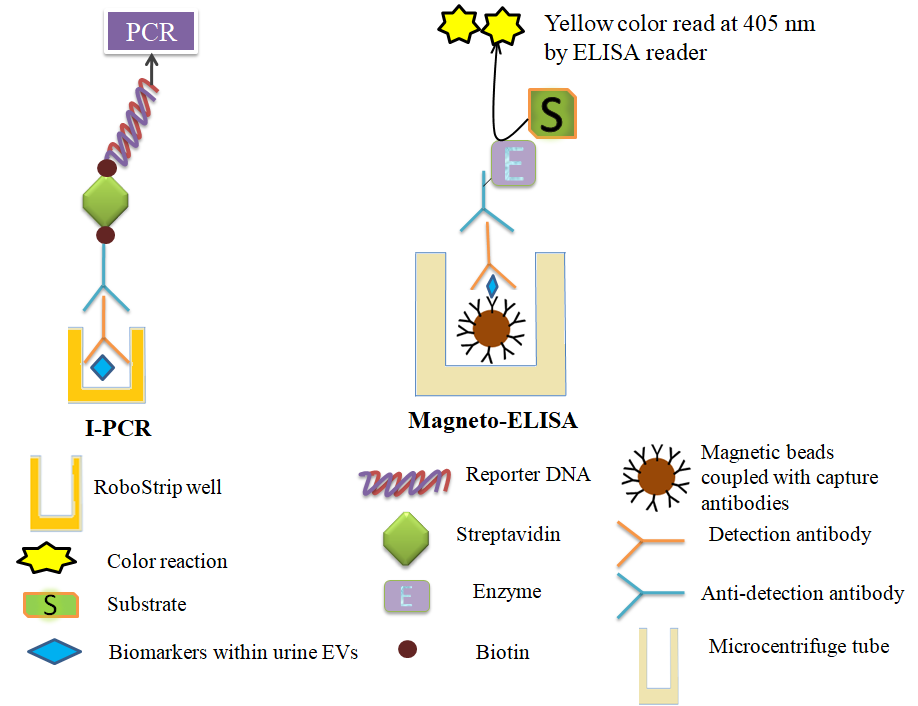


**Supplementary Figure 4 (b).** The schematic representation of I-PCR and Magneto-ELISA for *Mtb* LAM+MPT-64 detection within urine EVs of GUTB patients.

**
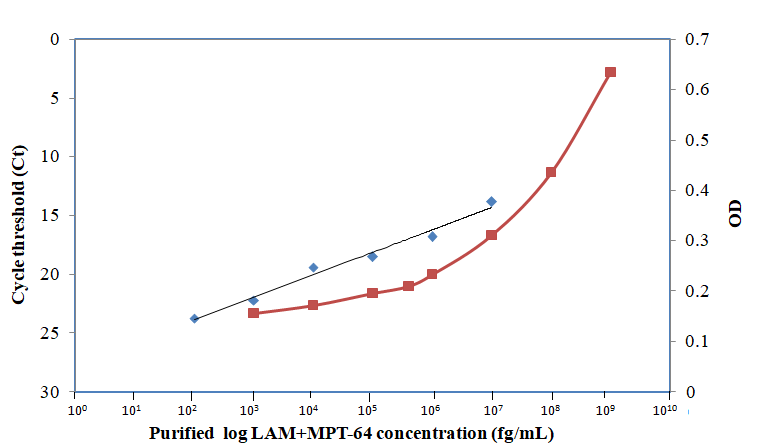
**

**Supplementary Figure 5.** The standard curve(s) for various dilutions of urine EVs (isolated from a healthy individual) spiked with purified LAM+MPT-64 (ranging from 100 fg/mL to 10 ng/mL) by MB-AuNP-RT-I-PCR and Magneto-ELISA (from 100 pg/mL to 1 µg/mL)**:** a correlation coefficient was found to be 0.98 for MB-AuNP-RT-I-PCR and the corresponding regression equation was Ct = -0.82 × ln (conc.) + 27.66. The mean Ct (for MB-AuNP-RT-I-PCR) and OD (for Magneto-ELISA) values were derived from two replicates. The experiments were repeated with spiked urine EVs (of another healthy individual) to ensure the authenticity of assays.


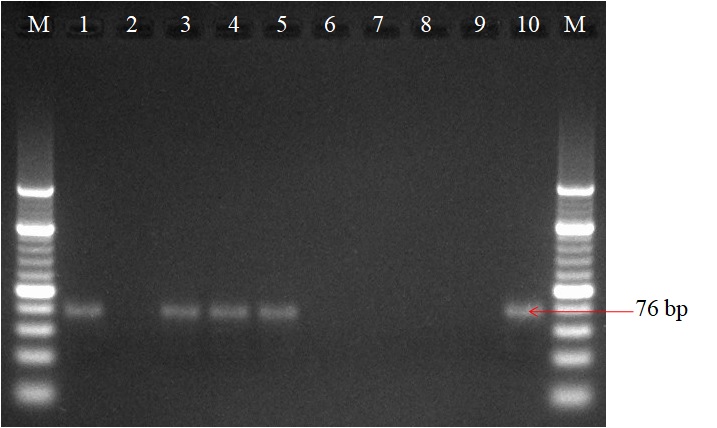


**Supplementary Figure 6.** Representative examples of GUTB cases and non-TB controls by MB-AuNP-I-PCR based on LAM+MPT-64 detection within urine EVs: Lane M, 20 bp ladder; lane 1, PCR-positive control (signal DNA, 1 ng/mL); lane 2, PCR-negative control (no template DNA); lanes 3-5, positive GUTB specimens; lanes 6-8, non-TB controls; lane 9, I-PCR-negative control (no antigen coated, rest all the reagents added); lane 10, I-PCR-positive control (purified LAM+MPT-64, 10 ng/mL).
