# Supplementary material for: Diagnosis of genitourinary tuberculosis: detection of mycobacterial lipoarabinomannan and MPT-64 biomarkers within urine extracellular vesicles by nano-based immuno-PCR assay
Source: Sci Rep. 2023 Jul 18;13:11560. doi: 10.1038/s41598-023-38740-3 (PMC10354090; doi:10.1038/s41598-023-38740-3)
Supplement: Supplementary file 2 — Supplementary Tables. [file 41598_2023_38740_MOESM2_ESM.docx]

**Supplementary Table 1. Replicates of ‘Mean Fluorescence Intensity’ values at different signal DNA concentrations from two independent experiments.**

| Signal DNA concentration (fmol/µL) | Experiment 1  Mean Fluorescence Intensity value (a.u.) | Experiment 2  Mean Fluorescence Intensity value (a.u.) | Final Mean Fluorescence Intensity value (a.u.) from Experiment 1 and Experiment 2 |
| --- | --- | --- | --- |
| 0 | 0.7055 | 0.7515 | 0.7285 |
| 0.5 | 43.839 | 47.1 | 45.4695 |
| 1 | 56.865 | 57.965 | 57.415 |
| 1.5 | 60.795 | 61.12 | 60.9575 |
| 2 | 83.22 | 86.315 | 84.7675 |

**Supplementary Table 2. Mean Ct and OD values for MB-AuNP-RT-I-PCR and Magneto-ELISA, respectively, from two independent experiments with different purified antigen (LAM+MPT-64) concentrations.**

|  | **MB-AuNP-RT-I-PCR** | | | **Magneto-ELISA** | | |
| --- | --- | --- | --- | --- | --- | --- |
| Ag (antigen) concentration | Mean Ct1 (from Experiment 1) | Mean Ct2 (from Experiment 2) | Final Mean Ct from Experiment 1 and Experiment 2 | Mean OD1 (from Experiment 1) | Mean OD2 (from Experiment 2) | Final Mean OD from Experiment 1 and Experiment 2 |
| 1 µg |  |  |  | 0.699 | 0.7465 | 0.72275 |
| 100 ng |  |  |  | 0.553 | 0.616 | 0.5845 |
| 10 ng | 9.2 | 9.31 | 9.255 | 0.356 | 0.4045 | 0.38025 |
| 1 ng | 13.9 | 13.5 | 13.7 | 0.252 | 0.285 | 0.2685 |
| 100 pg | 17.37 | 17.945 | 17.655 | 0.1925 | 0.194 | 0.19325 |
| 10 pg | 19.19 | 19.97 | 19.58 | 0.179 | 0.176 | 0.1775 |
| 1 pg | 21.065 | 21.835 | 21.4475 | 0.175 | 0.1715 | 0.17325 |
| 100 fg | 21.95 | 22.395 | 22.175 |  |  |  |
| Negative control (NC) | 23.62 | 24.605 | 24.1125 |  |  |  |
| Control (No Ag, rest all the reagents added) |  |  |  | 0.174 | 0.1805 | 0.17725 |

Based on NC+3SD (26.20) value, an LOD of 45 fg/mL was determined for the purified LAM+MPT-64 by MB-AuNP-RT-I-PCR. Likewise, based on mean OD of control + 2SD value (0.186), an LOD of 100 pg/mL was determined for the purified LAM+MPT-64 by Magneto-ELISA.
